# Biliousness

**Published:** 1903-07

**Authors:** J. A. Clement

**Affiliations:** Baltimore, Md.


### Biliousness.

BY J. A. CLEMENT, M. D., BALTIMORE, MD.

The world is full of biliousness. Now, I am free to admit that the term "biliousness" is not the least bit scientific, but the laity know what it means, and when they are given something to help it and that something does help it they want more of that something, which fact increases the demand for that something which, in this case, is Felsin.

I report results in four cases. In each one of these cases I used Felsin and nothing else. I wished to test the value of the preparation so that these cases show bona-fide results from the use of the tablets, and that is what physicians want:

Case 1.—Mr. G., age 40; occupation, patrolman. This patient is so situated that his meals are taken very irregularly and, as a rule, hurriedly. Natural result—dyspepsia. On January 18, 1903, called for prescription; complained of accumulation of much gas and rumbling in abdomen, pyrosis, bad taste in mouth, a sensation of "a rock in my stomach," bowels irregular and slight frontal headache. Gave Felsin tablets, one after each meal and one at bedtime. February 1st reported that he had received considerable relief. Continued the prescription. February 7th, reported all symptoms cleared up with the exception of the constipation, which was improving. Ordered Felsin, one tablet after meals, for an indefinite time.

Case 2.—Mr. M., age 53; no occupation. He belongs to what I term the "hot bird and cold bottle" class of dyspeptics. Imprudent both in eating and drinking. His main complaint was excessive flatulence, bilious headache and constipation. Gave Felsin tablets every four hours for three days, then one tablet after each meal and one at bedtime. The flatulence disappeared in two days, the headache in three, and the bowels are beginning to act as they should. If he will be careful with his diet and continue the use of the tablets for a couple of months I am confident he will get well, but such cases are hard to manage.

The tablets acted in this case very nicely, as, from the patient's description, he has about run through the list of digestives. Now he is grateful to Felsin.

Case 3.—Mrs. C., age 65. For three months past has noticed that if she takes anything more for supper than a cup of tea and a cracker or two she would awaken next morning with headache and more or less nausea. Also reported chronic constipation. Ordered Felsin tablets after each meal and on going to bed. The first two mornings after using the tablets awakened with headache, but no nausea; the third morning awakened free from both headache and nausea. Has now been using tablets two weeks and can eat a moderate meal at night with no bad result next day. Will continue use of tablets.

Case 4.—Mrs. D., age 24. Four months advanced in first pregnancy; complains that "everything she eats fills her full of wind and she can't get rid of it." Complaints of pregnant ladies must be taken with a grain of salt, but prescribed Felsin, one tablet after meals. After eight days treatment she reported that she had no more difficulty with the wind, but now she can't get enough to eat. Evidently Felsin in this case relieved the gas and stimulated the appetite.

[Editor's Note.—Felsin is a new preparation manufactured by the Viskolein Co., 210 Fulton Street, New York, who will no doubt send samples to physicians upon request.]

